# Long-term outcomes after ICU admission in critically ill patients with liver cirrhosis: An Australian state-wide cohort study

**DOI:** 10.1097/HC9.0000000000000762

**Published:** 2025-07-21

**Authors:** Danny Con, William Kemp, Avik Majumdar, David Pilcher, Stuart K. Roberts, Ammar Majeed

**Affiliations:** 1 Department of Gastroenterology, Austin Health, Melbourne, Victoria, Australia; 2 Department of Medicine, Faculty of Medicine, Dentistry and Health Sciences, The University of Melbourne, Victoria, Australia; 3 Department of Gastroenterology, Alfred Health, Melbourne, Victoria, Australia; 4 Faculty of Medicine, Nursing and Health Sciences, Central Clinical School, Monash University, Victoria, Australia; 5 Department of Intensive Care, Alfred Health, Melbourne, Victoria, Australia; 6 Centre for Outcomes and Resources Evaluation﻿, Australian and New Zealand Intensive Care Society, Prahran, Victoria, Australia; 7 Faculty of Medicine, Nursing and Health Sciences, Australian and New Zealand Intensive Care Research Centre, School of Public Health and Preventive Medicine, Monash University, Victoria, Australia

**Keywords:** chronic liver disease, cirrhotic, hepatic, transplant-free survival

## Abstract

**Background:**

Patients with liver cirrhosis requiring intensive care unit (ICU) admission have a high in-hospital mortality, yet long-term mortality and predictors of mortality in survivors remain unknown.

**Methods:**

All patients with liver cirrhosis admitted to 27 ICUs in Victoria, Australia, between 2007 and 2018 reported to the Australian and New Zealand Intensive Care Society Adult Patient Database were included. Poisson regression and Cox regression were used to explore factors associated with in-hospital mortality and all-cause 12-month mortality. Liver transplantation was considered a censoring event.

**Results:**

A total of 5725 cirrhosis patients (3565 patient-years) were included. All-cause 12-month mortality was 43.8% (95% CI 42.5–45.1) and reduced over time (HR 0.979 per year, 95% CI 0.967–0.991). On multivariable analysis, factors associated with in-hospital death (risk ratio—RR, 95% CI) included ACLF (acute-on-chronic liver failure) (1.73, 1.57–1.90), decompensated cirrhosis (1.31, 1.13–1.51), ALBI (albumin–bilirubin) grade 3 versus 2 (1.65, 1.45–1.87), APACHE-III ICU admission diagnosis of sepsis (1.43 vs. upper gastrointestinal bleeding, 1.24–1.66) or liver failure (1.81 vs. upper gastrointestinal bleeding, 1.55–2.12) and older age. On multivariable analysis of 4068 transplant-free hospital survivors, 12-month mortality was influenced by (HR, 95% CI) liver disease severity (1.37 ALBI grade 3 vs. 2, 1.15–1.64) and decompensated cirrhosis (1.25, 1.06–1.49) rather than ACLF (0.88, 0.75–1.03).

**Conclusions:**

Long-term mortality in liver cirrhosis patients admitted to the ICU is substantial but has decreased over time. ACLF confers a higher risk of in-hospital but not long-term death in hospital survivors. Cirrhosis severity and decompensation increase the risk of in-hospital and long-term mortality.

## INTRODUCTION

Liver cirrhosis represents a significant global health burden and is estimated to affect more than 100 million people worldwide.^1^^,^^2^ Liver cirrhosis confers a high mortality rate and contributes 2.4% of global deaths by previous estimates.^1^ Patients with liver cirrhosis are at increased risk of critical illness resulting from sepsis, variceal bleeding, and hepatic encephalopathy (HE)﻿.^2^^,^^3^ Multiorgan failure secondary to acute-on-chronic liver failure (ACLF) is also becoming increasingly recognized as a distinct pathological entity, contributing to a large proportion of intensive care unit (ICU) admissions in patients with cirrhosis and has a poor prognosis.^4^^–^^7^ Yet despite advances and optimization of ICU care, patients with liver cirrhosis admitted to the ICU have a substantial inpatient hospital mortality.^8^

Sepsis is well recognized as a major cause of hepatic decompensation in patients with cirrhosis, as well as a key trigger for ACLF.^9^ In-hospital mortality in patients with cirrhosis presenting with sepsis has improved over time, likely owing to improvements in sepsis recognition and management.^4^ In-hospital mortality of patients admitted with variceal bleeding has similarly decreased over time.^3^ However, long-term outcomes in critically ill patients with cirrhosis remain unknown. In particular, whether events such as sepsis or ACLF alter the trajectory of cirrhosis progression and increase the rate of long-term mortality remains unclear. Furthermore, in-hospital mortality due to ACLF has not mirrored the improvements in mortality due to sepsis and variceal bleeding, suggesting further research into ACLF management is required.^4^

The aims of this study were as follows: (1) to characterize all-cause 12-month mortality in patients with liver cirrhosis after admission to ICU, and to describe how this has changed over the study period; (2) to describe and explore risk factors associated with in-hospital mortality among patients with liver cirrhosis admitted to ICU; and (3) to describe and explore risk factors associated with 12-month mortality among ﻿hospital survivors.

## METHODS

The Australian and New Zealand Intensive Care Society Centre for Outcome and Research Evaluation (ANZICS CORE) Adult Patient Database (APD) contains information from over 2.5 million admissions to 212 ICUs in Australia and New Zealand, which represents over 90% of all ICU admissions in both countries and has previously been described.^10^ The study was conducted in accordance with the Declarations of Helsinki and Istanbul and approved by the Alfred Hospital Human Research Ethics Committee (project approval ID 732/19) in Melbourne, Australia, with a waiver for the requirement for informed consent.

### Study population

For the full cohort, all non-elective adult patients in the ANZICS-APD admitted to an ICU in the state of Victoria, Australia, between July 1, 2007, and December 31, 2018, who had a diagnosis of liver cirrhosis according to ANZICS-APD or by ICD codes were included (see Supplemental Tables S1 and S2, http://links.lww.com/HC9/C51). For patients who had multiple ICU admissions, only the first admission during the study period was included. Patients were excluded if they were admitted to the ICU following elective interventions or if they were admitted to the ICU for the purpose of receiving a liver transplantation. For the subcohort, patients were included if they survived their index hospital admission without requiring liver transplantation. A comparator non-cirrhosis cohort was identified to compare the 12-month mortality outcomes with the cirrhosis group. For this cohort, apart from the cirrhosis criteria, the same inclusion and exclusion criteria applied: only the first non-elective ICU admission was included, and admission for the purposes of liver transplantation was excluded.

### Data extraction

Data were extracted from the ANZICS-APD on unique patient identifiers, demographics, ICU admission diagnosis, vital signs and laboratory parameters on admission, year of admission, as well as in-ICU and in-hospital mortality. Only patients admitted to the ICU within the state of Victoria were included, as linkage data for long-term outcomes were only available for that state. The Victorian Admitted Episodes Dataset, a registry of all hospital admissions from the Government of Victoria, including all recorded International Classification of Diseases 10 (ICD-10) codes, was used to match ICD codes with the relevant admission for each patient (see Supplemental Table S2, http://links.lww.com/HC9/C51). The official Victorian government death registry (Births, Deaths and Marriages Victoria) was used to identify mortality of all included patients.

### Definitions

Patients with cirrhosis were identified using 2 methods: (1) flagged as having confirmed cirrhosis with portal hypertension by the ANZICS-APD; (2) presence of liver cirrhosis or decompensated cirrhosis using ICD codes (see Supplemental Table S2, http://links.lww.com/HC9/C51). ICD codes were used to identify complications of liver cirrhosis, including variceal bleeding, HE, and ascites. A Glasgow Coma Score (GCS) <15 was additionally used to identify patients with HE. Decompensated cirrhosis was defined as cirrhosis with additional history of variceal hemorrhage, ascites, and/or HE. ICD codes were also used to identify potential cirrhosis etiology, including alcohol-associated liver disease and viral liver diseases (see Supplemental Table S2, http://links.lww.com/HC9/C51).

ICU admission diagnosis was extracted from the ANZICS-APD, defined according to APACHE-III criteria, and grouped into 4 categories: liver failure, upper GI bleeding, sepsis, and other (see Supplemental Table S3, http://links.lww.com/HC9/C51). A diagnosis of ACLF was retrospectively applied using the NACSELD criteria,^4^^,^^11^ defined as liver decompensation with concurrent dysfunction of 2 or more extrahepatic organs as per data from the ANZICS-APD: brain failure was defined as GCS <15 on admission; respiratory failure was defined as presence of assisted ventilation (invasive or noninvasive); renal failure was defined as either requirement of renal replacement therapy, serum creatinine ≥350 µmol/L and/or 24-hour urine output <500 mL as surrogates for need for renal replacement therapy; circulatory failure was defined as mean arterial pressure <60 mm Hg and/or requirement of ionotropic support. The albumin–bilirubin grade (ALBI) was calculated as ALBI=log_10_[0.66×bilirubin µmol/L]–0.085×[albumin g/L]^12^ and used as a measure of liver function. The MELD score to assess liver function could not be derived as the ANZICS-APD does not collect information on international normalized ratio (INR).

### Outcomes

The primary outcome of interest was all-cause mortality at 12 months after ICU admission. We also examined in-hospital mortality after ICU admission and 12-month mortality in hospital survivors.

### Statistical analysis

Continuous variables were expressed as medians with IQR, while categorical variables were expressed as frequencies and percentages. No imputation for missing data was performed, and the proportion of missing data for each variable is provided. Logistic regression was used to determine whether the proportion of admissions with liver cirrhosis, decompensated cirrhosis, or ACLF changed over time. Modified Poisson regression was used to estimate risk ratios (RRs) and confidence intervals (﻿CIs) for in-hospital mortality.^13^ In transplant-free hospital survivors, Cox proportional hazards regression was used to estimate hazard ratios (﻿HRs) and CIs for 12-month mortality. Patients entered the risk set upon discharge from the hospital. Patients were censored at 12 months or at the time of liver transplantation if this occurred prior to 12 months. The effects of patient demographics, liver compensation status, presence of ACLF, cirrhosis etiology, ALBI grade, and principal ICU admission diagnosis on mortality were﻿ explored using both univariable and multivariable regression. To avoid collinearity in the multivariable regression models, a modified Charlson comorbidity index, excluding age and liver categories, was used in conjunction with a separate age variable and ALBI grade. The Kaplan–Meier method was used to plot the survival function over time. A 2-sided *p*<0.05 was considered to be statistically significant. All analyses were performed using R version 4.2.2 (RStudio).

## RESULTS

### Baseline demographics

**FIGURE 1:**
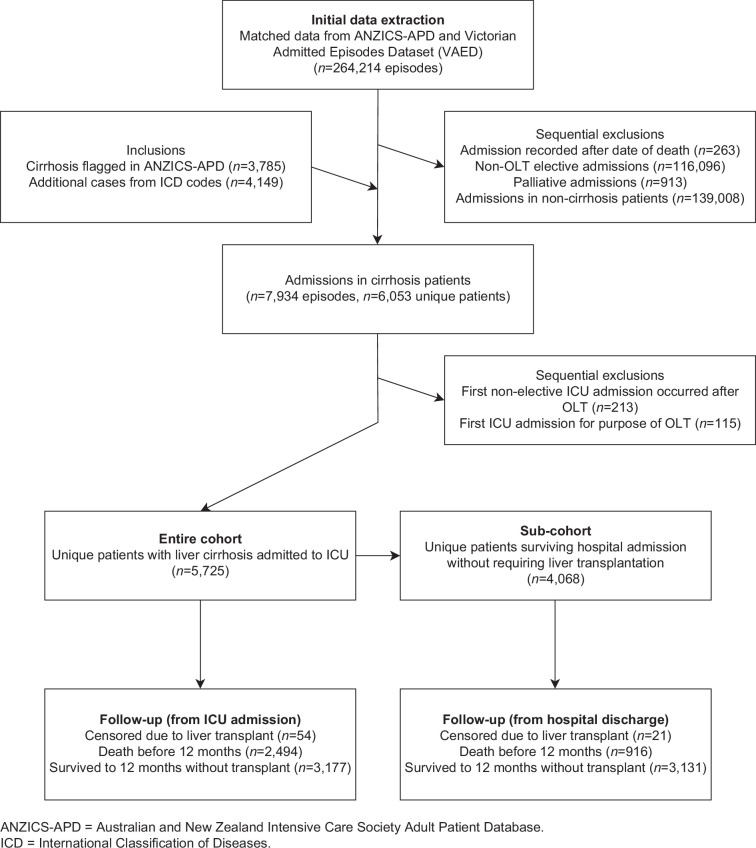
Flow diagram of data extraction. Abbreviations: ANZICS-APD, Australian and New Zealand Intensive Care Society Adult Patient Database; ICD, International Classification of Diseases; OLT, orthotopic liver transplantation.

**FIGURE 2:**
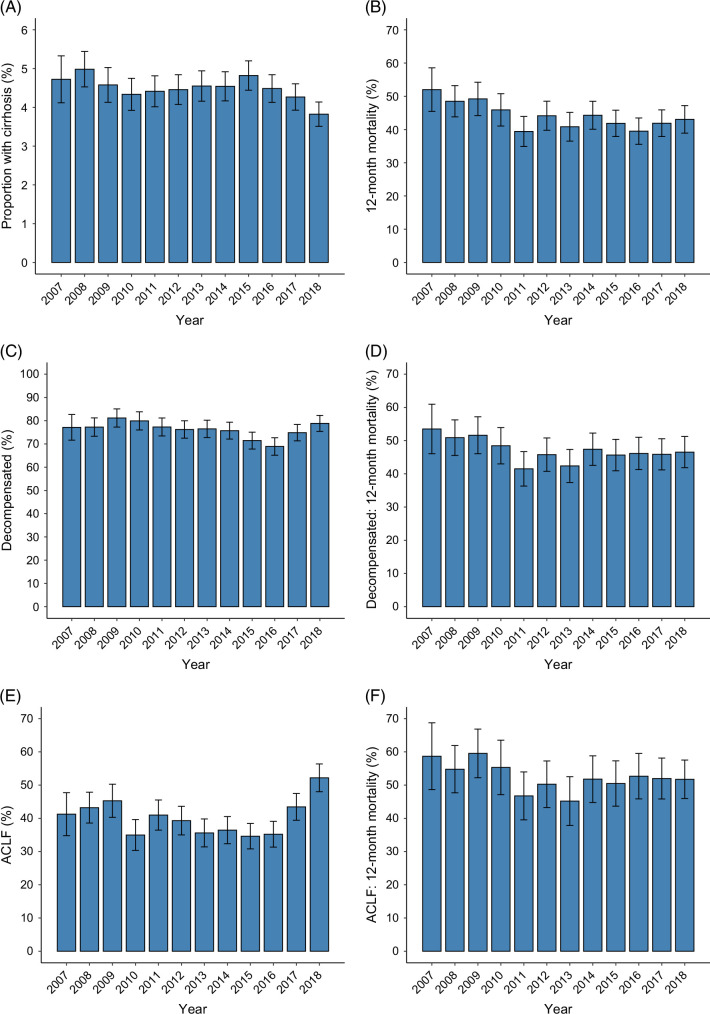
Characteristics and mortality of cirrhosis patients admitted to the ICU over time, with error bars showing 95% CIs. (A) Percentage of all ICU admissions that had liver cirrhosis (reduction over time; OR 0.988 per year, 95% CI 0.980–0.995, *p*=0.0021). (B) Twelve-month mortality in cirrhosis patients, reduction over time (HR 0.979 per year, 95% CI 0.967–0.991, *p*=0. 0 0055). (C) Proportion of cirrhosis patients with decompensation, showing overall decrease over time (OR 0.972 per year, 95% CI 0.955–0.991, *p*=0.0 038). (D) Twelve-month mortality in decompensated cirrhosis patients, showing no significant change over time (HR 0.989 per year, 95% CI 0.976–1.002, *p*=0.088). (E) Proportion of cirrhosis patients with ACLF, showing no change over time (OR 1.009 per year, 95% CI 0.993–1.025, *p*=0.27). (F) Twelve-month mortality among ACLF patients, showing no significant change over time (HR 0.987, 95% CI 0.971–1.003, *p*=0.12). Abbreviations: ACLF, acute-on-chronic liver failure; ICU, intensive care unit.

A total of 5725 unique patients with liver cirrhosis admitted non-electively to the ICU from 27 Victorian hospitals were included in the study, with a total follow-up of 3565 patient-years (accounting for censoring at 12 months) (see Figure 1). The mean age was 58.9 years (SD 13.9), and 36.9% were female (see Table 1). Etiology of liver cirrhosis included alcohol (47.4%), viral hepatitis (22.2%) (not mutually exclusive), and other (43.8%). Patients were hospitalized prior to ICU admission for a median of 8.7 hours (IQR 3.2–49.2). Median ICU length of stay was 75.0 hours (IQR 38.1–164.4). Overall, 5414 (94.6%) had portal hypertension, 4342 (75.8%) had decompensated cirrhosis, and 2294 (40.1%) had ACLF. In total, 77.9% had an ALBI grade of 3, while 21.0% had an ALBI grade of 2, and only 1.1% had an ALBI grade of 1. Patients with liver cirrhosis represented 4.5% of all non-elective unique ICU admissions (*n*=128,308) during the same period. There was a significant decrease in the proportion of ICU patients admitted who had liver cirrhosis over time (OR 0.988 per year, 95% CI 0.980–0.995, *p*=0.0021) (see Figure 2), although there was an overall increase in the absolute number of cirrhosis patients admitted to ICU over the same period (6.5% increase per year, 95% CI 3.7%–9.3%, *p*=0.0009).

**TABLE 1:** Baseline characteristics of the full cohort (all patients admitted to the ICU with liver cirrhosis; *n*=5725) and the subcohort (subset of patients who survived the ICU and hospital admission without requiring liver transplantation; *n*=4068)

	Full cohort: all patients admitted to the ICU		Subcohort: survivors of hospital admission	
Variable	*n*	Frequency (%)	*n*	Frequency (%)
Age, y, mean±SD	5725	58.9±13.9	4068	58.0±14.1
Age category	5725		4068	
<50 y		1396 (24.4)		1080 (26.5)
50–59 y		1526 (26.7)		1103 (27.1)
60–69 y		1472 (25.7)		1009 (24.8)
70–79 y		943 (16.5)		632 (15.5)
≥80 y		388 (6.8)		244 (6.0)
Sex	5725		4068	
Male		3610 (63.1)		2544 (62.5)
Female		2115 (36.9)		1524 (37.5)
Charlson comorbidity index, median (IQR)	5725	5 (4–7)	4068	5 (3–7)
Cirrhosis etiology: alcohol	5725	2712 (47.4)	4068	1934 (47.5)
Cirrhosis etiology: viral	5725	1273 (22.2)	4068	902 (22.2)
Presence of portal hypertension	5725	5414 (94.6)	4068	3813 (93.7)
Cirrhosis status during hospital admission	5725		4068	
Compensated		1383 (24.2)		1145 (28.1)
Decompensated		4342 (75.8)		2923 (71.9)
Presence of ACLF during ICU admission	5725	2294 (40.1)	4068	1361 (33.5)
Length of stay prior to ICU admission, hours, median (IQR)	5681	8.7 (3.2–49.2)	4065	7.9 (3.1–33.0)
Principal ICU admission diagnosis	5725		4068	
Liver failure		666 (11.6)		382 (9.4)
Upper GI bleeding		895 (15.6)		703 (17.3)
Sepsis		1358 (23.7)		885 (21.8)
Renal failure		251 (4.4)		173 (4.3)
Respiratory-related		243 (4.2)		173 (4.3)
Cardiac-related		549 (9.6)		347 (8.5)
Neurological-related		566 (9.9)		445 (10.9)
Malignancy-related		125 (2.2)		89 (2.2)
Trauma		179 (3.1)		149 (3.7)
Gastrointestinal (excl. upper GI bleeding)		568 (9.9)		460 (11.3)
Hematological-related		52 (0.9)		28 (0.7)
Other		273 (4.8)		234 (5.8)
Length of ICU stay, hours, median (IQR)	5723	75.0 (38.1–164.4)	4097	70.6 (37.5–144.3)
Assisted ventilation	5704	2454 (43.0)	4051	1562 (38.6)
ALBI, mean±SD	5280	−0.81±0.76	3745	−0.91±0.73
ALBI grade	5280		3745	
Grade 1		58 (1.1)		48 (1.3)
Grade 2		1109 (21.0)		895 (23.9)
Grade 3		4113 (77.9)		2802 (74.8)
Laboratory markers, median (IQR)				
Hemoglobin, g/dL	3968	9.3 (7.9–11.0)	2827	9.4 (8.0–11.1)
Platelet count, ×10^9^/L	3944	113.0 (68.0–189.0)	2813	118.0 (71.0–195.0)
Sodium, mmol/L	5643	136.0 (131.0–139.0)	4039	136.0 (132.0–139.0)
Creatinine, µmol/L	5576	107.0 (62.0–196.0)	4000	90.0 (59.0–166.0)
Bilirubin, µmol/L	5335	30.0 (14.0–69.0)	3805	26.0 (13.0–55.0)
Albumin, g/L	5487	25.0 (21.0–29.0)	3930	25.0 (21.0–30.0)

*Note*: ACLF was defined using NACSELD criteria, defined as liver decompensation with concurrent dysfunction of 2 or more extrahepatic organs as per data from the ANZICS-APD.

Abbreviations: ACLF, acute-on-chronic liver failure; ALBI, albumin–bilirubin score; ICU, intensive care unit.

### Overall mortality and liver transplantation

**FIGURE 3:**
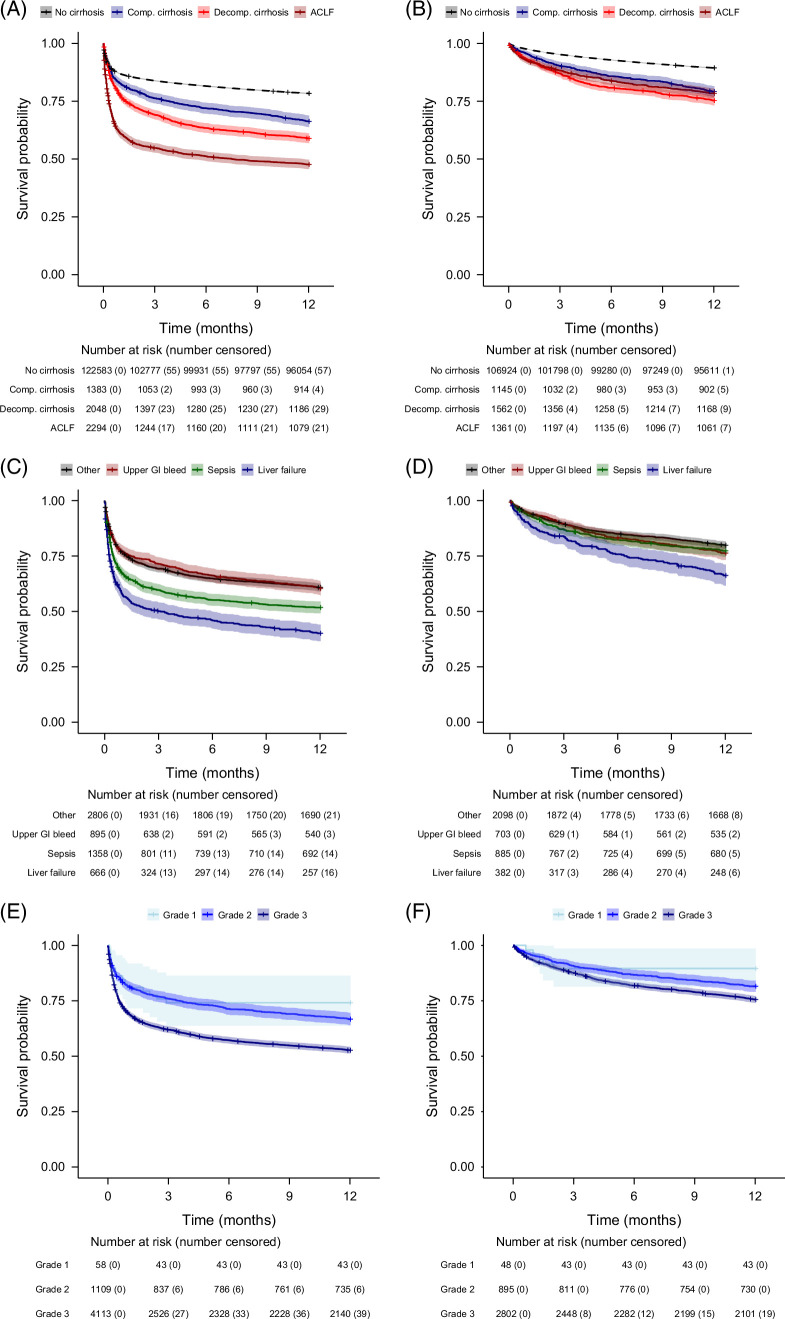
Kaplan–Meier plots of mortality of all patients non-electively admitted to the ICU and in transplant-free hospital survivors. (A) All patients stratified by cirrhosis status. (B) Transplant-free hospital survivors stratified by cirrhosis status. (C) All patients stratified by APACHE-III admission diagnosis. (D) Transplant-free hospital survivors stratified by APACHE-III admission diagnosis. (E) All patients stratified by ALBI grade. (F) Transplant-free hospital survivors stratified by ALBI grade. Abbreviations: ACLF, acute-on-chronic liver failure; ALBI, albumin–bilirubin; Comp., compensated; Decomp., decompensated; GI, gastrointestinal; ICU, intensive care unit.

Among patients with liver cirrhosis, 1627 (28.4%) died in hospital while 4098 (71.6%) survived. The proportion who died in hospital was higher among patients with cirrhosis compared to those without cirrhosis (12.7%, *p*<0.0001). Fifty-four (0.9%) patients received a liver transplant within the first year after their index ICU admission. The overall 1, 3, and 12 months mortality rates in patients with cirrhosis were 27.9% (95% CI 26.7–29.1), 34.8% (95% CI 33.6–36.1), and 43.8% (95% CI 42.5–45.1), respectively. The corresponding 1, 3, and 12 months mortality rates in non-elective patients without cirrhosis were 13.2% (95% CI 13.0–13.4), 16.1% (95% CI 15.9–16.3), and 21.6% (95% CI 21.4–21.9), respectively. Patients with compensated cirrhosis had the lowest in-hospital mortality (238/1383, 12.7%), which was lower than decompensated cirrhosis patients without ACLF (468/2048, 22.9%) and ACLF patients (921/2294, 40.1%) (see Figure 3).

There was a reduction in overall mortality over time when considering all patients with cirrhosis (HR 0.979 per year, 95% CI 0.967–0.991, *p*=0.00055), possibly owing to a reduction in the proportion of patients admitted with decompensated cirrhosis over time (OR 0.972 per year, 95% CI 0.955–0.991, *p*=0.0038). However, there was no significant change in overall mortality over time in patients with decompensated cirrhosis (HR 0.989 per year, 95% CI 0.976–1.002, *p*=0.088) or in patients with ACLF (HR 0.987, 95% CI 0.971–1.003, *p*=0.12), and the proportion admitted with ACLF did not change significantly over time (OR 1.009 per year, 95% CI 0.993–1.025, *p*=0.27) (see Figure 2).

### Factors associated with in-hospital mortality

On univariable analysis (Table 2), patients with liver cirrhosis had a higher in-hospital mortality if they were older (risk ratio—RR 1.013 per year, 95% CI 1.010–1.016), had a higher Charlson comorbidity index (RR 1.11 per point, 95% CI 1.10–1.13), had decompensated cirrhosis (30.0% mortality in decompensated cirrhosis patients vs. 17.2% in compensated cirrhosis patients; RR 1.86, 95% CI 1.64–2.10) or ACLF (40.1% mortality in ACLF patients vs. 20.6% in non-ACLF patients; RR 1.95, 95% CI 1.80–2.12). Compared to patients who presented with an APACHE-III principal ICU admission diagnosis of upper gastrointestinal bleeding, in-hospital mortality was higher in patients who had a principal diagnosis of sepsis (RR 1.60, 95% CI 1.39–1.86) or liver failure (RR 1.94, 95% CI 1.66–2.27) (see Figure 3). Patients were more likely to die in the hospital if they had a higher ALBI grade (RR 1.68 grade 3 vs. 2, 95% CI 1.47–1.91) (see Figure 3). Neither viral hepatitis nor alcohol as contributing factors to cirrhosis etiology were associated with in-hospital mortality risk.

**TABLE 2:** Estimated risk ratios and 95% CIs for in-hospital death among patients with cirrhosis admitted to the ICU (*n*=5725) using modified Poisson regression

	Univariable		Multivariable model 1		Multivariable model 2	
Variable	RR (95% CI)	*p*	RR (95% CI)	*p*	RR (95% CI)	*p*
Age, per year	1.013 (1.010–1.016)	<0.0001	1.015 (1.012–1.018)	<0.0001	—	—
Age category						
<50 y	1.00 (reference)	—	—	—	1.00 (reference)	—
50–59 y	1.21 (1.06–1.37)	0.0040	—	—	1.17 (1.03–1.33)	0.014
60–69 y	1.40 (1.24–1.59)	<0.0001	—	—	1.32 (1.16–1.49)	<0.0001
70–79 y	1.48 (1.29–1.69)	<0.0001	—	—	1.44 (1.25–1.65)	<0.0001
≥80 y	1.68 (1.43–1.97)	<0.0001	—	—	1.72 (1.46–2.04)	<0.0001
Male (vs. female)	1.06 (0.98–1.16)	0.16	1.05 (0.96–1.14)	0.28	1.06 (0.97–1.15)	0.19
CCI, per point	1.11 (1.10–1.13)	<0.0001	—	—	—	—
CCI, excluding age and liver disease, per point	1.11 (1.09–1.13)	<0.0001	1.10 (1.08–1.12)	<0.0001	1.10 (1.08–1.12)	<0.0001
Cirrhosis etiology: alcohol	0.98 (0.90–1.06)	0.61	0.98 (0.90–1.06)	0.60	0.97 (0.89–1.05)	0.42
Cirrhosis etiology: viral	1.01 (0.92–1.12)	0.82	1.01 (0.92–1.11)	0.89	1.00 (0.91–1.10)	1.0
Cirrhosis status during hospital admission						
Compensated	1.00 (reference)	—	1.00 (reference)	—	1.00 (reference)	—
Decompensated	1.86 (1.64–2.10)	<0.0001	1.31 (1.14–1.51)	0.00014	1.31 (1.13–1.51)	0.00024
Presence of ACLF during ICU admission	1.95 (1.80–2.12)	<0.0001	1.67 (1.52–1.84)	<0.0001	1.73 (1.57–1.90)	<0.0001
Principal ICU admission diagnosis						
Upper gastrointestinal bleed	1.00 (reference)	—	1.00 (reference)	—	1.00 (reference)	—
Sepsis	1.60 (1.39–1.86)	<0.0001	1.45 (1.25–1.67)	<0.0001	1.43 (1.24–1.66)	<0.0001
Liver failure	1.94 (1.66–2.27)	<0.0001	1.70 (1.46–1.98)	<0.0001	1.81 (1.55–2.12)	<0.0001
Other	1.16 (1.01–1.34)	0.039	1.26 (1.10–1.44)	0.0010	1.15 (1.00–1.32)	0.059
ALBI, per unit	1.56 (1.47–1.65)	<0.0001	1.59 (1.49–1.69)	<0.0001	—	—
ALBI category						
Grade 1	0.92 (0.52–1.64)	0.77	—	—	0.95 (0.55–1.65)	0.86
Grade 2	1.00 (reference)	—	—	—	1.00 (reference)	—
Grade 3	1.68 (1.47–1.91)	<0.0001	—	—	1.65 (1.45–1.87)	<0.0001

Model 1: multivariable model utilizing continuous variables.

Model 2: multivariable model utilizing categorical variables for age and ALBI grade.

Abbreviations: ACLF, acute-on-chronic liver failure; ALBI, albumin–bilirubin score; CCI, Charlson comorbidity index; ICU, intensive care unit; RR, risk ratio.

On multivariable analysis (Table 2), ACLF conferred the highest risk of in-hospital death, where patients with ACLF had an 73% higher risk of death compared to non-ACLF patients (RR 1.73, 95% CI 1.57–1.90), while patients with decompensated liver cirrhosis had a 31% higher risk of death (RR 1.31, 95% CI 1.13–1.51). Patients aged 80 years or greater were 72% more likely to die in hospital compared to those aged under 50 years (RR 1.72, 95% CI 1.46–2.04). Compared to patients admitted with an APACHE-III principal diagnosis of upper gastrointestinal bleeding, patients admitted with a principal diagnosis of sepsis had a 43% higher risk of death (RR 1.43, 95% CI 1.24–1.66) and patients with a principal diagnosis of liver failure had an 81% higher risk of death (RR 1.81, 95% CI 1.55–2.12). Patients with ALBI grade 3 were 65% more likely to die in hospital compared to ALBI grade 2 (RR 1.65, 95% CI 1.45–1.87).

### Demographics of transplant-free hospital survivors

Of 4098 patients with liver cirrhosis who survived the index hospital admission, 30 (0.7%) underwent liver transplantation during the admission and were excluded from the cohort of transplant-free hospital survivors. Of the 4068 included patients, the mean age was 58.0 years (SD 14.1), and 37.5% were female (Table 1). Of these, 3813 (93.7%) had portal hypertension, 2923 (71.9%) had decompensated cirrhosis, and 1361 (33.5%) had ACLF during the index admission. Overall, 74.8% had an ALBI grade of 3, 23.9% had an ALBI grade of 2, and only 1.3% had an ALBI grade of 1. In this subcohort, 21 (0.5%) patients underwent subsequent liver transplantation within 12 months, and the 1, 3, and 12 months (transplant-excluded) mortality rates were 6.1% (95% CI 5.4–6.9), 11.6% (95% CI 10.6–12.6), and 22.6% (95% CI 21.3–23.8), respectively.

### Factors associated with long-term mortality among hospital survivors

On univariable analysis of hospital survivors (Table 3), the 12-month mortality risk was estimated to be higher in patients who were older (HR 1.022 per year, 95% CI 1.017–1.026), were male (HR 1.37 vs. female, 95% CI 1.19–1.58), had greater comorbidities (HR 1.18 per CCI point, 95% CI 1.16–1.21) and had higher ALBI grade (HR 1.37 grade 3 vs. 2, 95% CI 1.15–1.64). Decompensated cirrhosis status during the hospital admission was estimated to confer a 15% increase in 12-month mortality risk, which was not statistically significant (HR 1.15, 95% CI 0.99–1.33). Compared to patients whose index ICU admission APACHE-III principal diagnosis had been upper gastrointestinal bleeding, the 12-month mortality risk was higher in patients who had a principal admission diagnosis of liver failure (HR 1.53, 95% CI 1.22–1.93), but not sepsis (HR 0.97, 95% CI 0.79–1.19) (see Figure 3).

**TABLE 3:** Estimated HRs and 95% CIs for 12-month mortality among patients with cirrhosis surviving index ICU and hospital admission (*n*=4068) using Cox proportional hazards regression

	Univariable		Multivariable model 1		Multivariable model 2	
Variable	HR (95% CI)	*p*	HR (95% CI)	*p*	HR (95% CI)	*p*
Age, per year	1.022 (1.017–1.026)	<0.0001	1.023 (1.017–1.028)	<0.0001	—	—
Age category						
<50 y	1.00 (reference)	—	—	—	1.00 (reference)	—
50–59 years	1.49 (1.22–1.82)	<0.0001	—	—	1.34 (1.09–1.64)	0.0061
60–69 years	1.74 (1.43–2.12)	<0.0001	—	—	1.50 (1.21–1.85)	0.00015
70–79 years	2.09 (1.69–2.58)	<0.0001	—	—	1.94 (1.55–2.43)	<0.0001
≥80 years	2.20 (1.67–2.90)	<0.0001	—	—	2.13 (1.58–2.86)	<0.0001
Male (vs. female)	1.37 (1.19–1.58)	<0.0001	1.31 (1.13–1.52)	0.00026	1.33 (1.14–1.53)	0.00017
CCI, per point	1.18 (1.16–1.21)	<0.0001	—	—	—	—
CCI, excluding age and liver disease, per point	1.23 (1.19–1.27)	<0.0001	1.22 (1.18–1.27)	<0.0001	1.22 (1.18–1.27)	<0.0001
Cirrhosis etiology: alcohol	1.05 (0.93–1.20)	0.42	0.99 (0.86–1.13)	0.85	0.99 (0.86–1.13)	0.88
Cirrhosis etiology: viral	1.16 (1.00–1.35)	0.052	1.11 (0.94–1.29)	0.21	1.11 (0.95–1.30)	0.20
Cirrhosis status during hospital admission						
Compensated	1.00 (reference)	—	1.00 (reference)	—	1.00 (reference)	—
Decompensated	1.15 (0.99–1.33)	0.062	1.26 (1.07–1.50)	0.0071	1.25 (1.06–1.49)	0.0095
Presence of ACLF during ICU admission	0.94 (0.82–1.08)	0.36	0.87 (0.74–1.01)	0.073	0.88 (0.75–1.03)	0.11
Principal ICU admission diagnosis						
Upper gastrointestinal bleed	1.00 (reference)	—	1.00 (reference)	—	1.00 (reference)	—
Sepsis	0.97 (0.79–1.19)	0.77	0.84 (0.68–1.04)	0.10	0.84 (0.68–1.04)	0.11
Liver failure	1.53 (1.22–1.93)	0.00028	1.63 (1.28–2.08)	<0.0001	1.68 (1.32–2.13)	<0.0001
Other	0.84 (0.70–1.01)	0.063	0.78 (0.64–0.95)	0.012	0.74 (0.61–0.90)	0.0028
ALBI, per unit	1.33 (1.21–1.46)	<0.0001	1.42 (1.28–1.57)	<0.0001	—	—
ALBI category						
Grade 1	0.55 (0.23–1.35)	0.19	—	—	0.64 (0.26–1.56)	0.33
Grade 2	1.00 (reference)	—	—	—	1.00 (reference)	—
Grade 3	1.38 (1.16–1.63)	0.00023	—	—	1.37 (1.15–1.64)	0.00010

Model 1: multivariable model utilizing continuous variables.

Model 2: multivariable model utilizing categorical variables for age and ALBI grade.

Abbreviations: ACLF, acute-on-chronic liver failure; ALBI, albumin–bilirubin score; CCI, Charlson comorbidity index; ICU, intensive care unit.

On multivariable analysis of hospital survivors, longer-term mortality was influenced by compensation status (decompensated vs. compensated HR 1.25, 95% CI 1.06–1.49) and liver disease severity (ALBI grade 3 vs. 2 HR 1.37, 95% CI 1.15–1.64) but not ACLF status at the time of hospitalization (ACLF vs. no-ACLF HR 0.88, 95% CI 0.75–1.03) (see Table 3 and Figure 3). The observed 12-month mortality rate in patients who survived the hospital admission was 10.6% (11,331/106,924) in non-cirrhosis patients, 20.8% (238/1145) in patients with compensated cirrhosis during hospital admission, 24.6% (385/1562) in decompensated cirrhosis patients without ACLF and 21.5% (293/1361) in patients who had ACLF during hospital admission. Compared to patients who had an APACHE-III principal ICU admission diagnosis of upper gastrointestinal bleeding, the estimated risk of death within 12 months was 68% higher in patients who had an admission diagnosis of liver failure (HR 1.68, 95% CI 1.32–2.13), but estimated to be 26% lower in patients admitted with non-liver and non-sepsis related reasons (HR 0.74, 95% CI 0.61–0.90), and not significantly different compared to patients admitted with a principal diagnosis of sepsis (see Figure 3). Older age and male sex remained significantly associated with an increased risk of death within 12 months on multivariable analysis. Neither alcohol nor viral hepatitis, as contributing factors to cirrhosis etiology, affected mortality.

## DISCUSSION

In this large state-wide cohort involving 5725 non-elective ICU admissions in patients with liver cirrhosis, we found that both short-term and long-term mortality were high, where more than a quarter of patients did not survive their index hospital admission, and more than two-fifths died by 12 months. A majority of patients had decompensated liver disease, while 40% had ACLF. Overall, 12-month mortality decreased over time in patients with liver cirrhosis, which is similar to previous findings.^14^^,^^15^ The proportion of cirrhosis patients admitted to the ICU with liver decompensation decreased over time, while the proportion with ACLF remained stable over time. A key finding was that although in-hospital mortality was expectedly driven by both ACLF status and compensation status, longer-term mortality in hospital survivors was influenced by compensation status but not ACLF status. ICU admission due to liver failure confers the greatest risk of death over admission due to sepsis, upper GI bleeding and non-liver related reasons. A higher ALBI grade, older age, and greater Charlson comorbidity index increased the risk of both in-hospital and 12-month mortality.

Understanding longer-term mortality after hospital discharge remains key in the management of patients with liver cirrhosis.^3^^,^^4^ The presence of ACLF using the NACSELD definition conferred a substantially increased risk of in-hospital mortality. However, in patients who survived their index hospital admission and recovered from ACLF, the risk of death up to 12 months was no higher than in patients who did not have ACLF. Despite this, mortality after ACLF remains high, and rates of liver transplantation remain low (0.9% in our study compared to 3.8% from a previous meta-analysis^16^), which suggests a need for better access to liver transplantation for patients with ACLF. In contrast, compensation status at the time of ICU admission does influence the risk of death even after discharge, which is an expected outcome. Our findings are similar to a recent Dutch cohort study, which found that ACLF grade was not found to be independently associated with 12-month survival after hospital discharge.^17^ However, our findings do differ from a South Korean cohort study that found that while ACLF did not affect long-term survival in compensated patients after recovery, ACLF did worsen survival in decompensated patients.^18^ Therefore, it remains to be seen whether ACLF is truly a reversible insult that does not confer an additional residual risk of death after a full recovery is made, given the provision of proper ICU and hospital care.

We also found that ICU admission diagnosis plays an important role in determining long-term mortality risk. Patients admitted with a principal ICU admission diagnosis of liver failure had the highest in-hospital and long-term mortality, which is an expected finding, while sepsis as the reason for ICU admission conferred the second-highest in-hospital mortality risk after liver failure. Up to one-third of ICU admissions in patients with cirrhosis is related to sepsis and its complications.^19^ Sepsis is not only more common in patients with cirrhosis compared to the general population, but is also more severe and is associated with a 4-fold increase in mortality.^20^^,^^21^ It is also one of the commonest triggers of ACLF.^5^^,^^9^ However, patients admitted for sepsis who survived their hospital admission did not have a significantly higher risk of death at 12 months compared to patients admitted for non-sepsis and non-liver related reasons. This may be partly explained by the higher proportion of patients with sepsis who had ACLF (41.0%) compared to patients presenting with upper GI bleeding (34.8%). This suggests that while sepsis is a serious complication in patients with cirrhosis, overall long-term mortality can be mitigated by appropriate in-hospital and post-discharge sepsis management and prevention.

In-hospital mortality in patients admitted with a principal ICU diagnosis of upper GI bleeding was lower than other causes, which is similar to a previous meta-analysis.^16^ However, in contrast to the association between sepsis and mortality, patients who survived their admission for upper GI bleeding had a similar long-term prognosis as patients admitted for sepsis. This suggests that while acute variceal bleeding can be well treated in the hospital with medical, endoscopic, and interventional radiologic approaches,^22^^–^^24^ secondary prevention after variceal bleeding may not be sufficient alone to influence longer-term survival, although our data did not capture such methods of secondary prevention.

The strengths of our study include the sample size, where our study examines a large cohort of cirrhosis patients admitted to the ICU, as well as the subcohort of patients who survived their index hospital admission. Furthermore, we were able to extract additional variables using admission-based ICD codes to further enrich the prospective ANZICS database. However, our study had several limitations. There is a risk of information bias due to misclassification. Our assumption for identifying HE in patients with proven portal hypertension using GCS <15 may have falsely classified patients who had reduced GCS due to other causes, and therefore overestimated the true proportion with HE﻿, cirrhosis decompensation, and ACLF. Also, raised creatinine and oliguria were used as surrogates for the need for renal replacement therapy in the definition for ACLF. Despite this, the proportion of patients in our cohort with ACLF appeared lower than in a recent study where 88% of cirrhosis patients admitted to the ICU experienced ACLF, and may reflect our expanded criteria allowing the inclusion of cirrhosis patients without prior decompensation.^17^ Results may not be generalizable to localities with different demographic profiles and treatment practices. A major limitation is the lack of INR data, which prohibits the calculation and analysis of the MELD scores. However, this was mitigated to an extent by the inclusion of liver decompensation as an explanatory variable, as well as the use of ALBI grade. The study was limited by the inability to further characterize the cause of death, and patients who died outside of Victoria would not have been captured.

In conclusion, through robust methodology, we have characterized the longer-term trends in mortality in patients with liver cirrhosis after admission to the ICU and explored risk factors of in-hospital mortality and 12-month mortality after hospital discharge. Baseline hepatic compensation status, rather than ACLF, most influences long-term mortality in hospital survivors. Sepsis is a poor prognostic factor and is associated with a high in-hospital mortality; however, longer-term mortality is mitigated after surviving the index admission. Factors associated with poorer outcomes can be incorporated into prognostication tools in future research.

## Supplementary Material
