# Supplementary material for: Long-term outcomes after ICU admission in critically ill patients with liver cirrhosis: An Australian state-wide cohort study
Source: Hepatol Commun. 2025 Jul 21;9(8):e0762. doi: 10.1097/HC9.0000000000000762 (PMC12282839; doi:10.1097/HC9.0000000000000762)
Supplement: Supplementary file 1 [file hc9-9-e0762-s001.pdf]

SUPPLEMENTARY TABLES

Table of Contents

Page 2 ……… Supplementary Table 1. Definition of cirrhosis categories.

Page 3 ……… Supplementary Table 2. ICD-10 codes used to define complications of cirrhosis and cirrhosis aetiology.

Page 4 ……… Supplementary Table 3. APACHE-III diagnosis codes used to define ICU admission diagnosis categories and liver transplantation.

Supplementary Table 1. Definition of cirrhosis categories.

Variable	Study definition
Cirrhosis	*ANZICS-APD flagged cirrhosis* OR *ICD-10 flagged cirrhosis.*
ANZICS-APD flagged cirrhosis	Proven cirrhosis and documented portal hypertension; or episodes of past upper GI bleed attributed to portal hypertension. If the patient has a functioning liver transplant, this chronic health item does not apply.†
ICD-10 flagged cirrhosis	*Varices* OR *variceal bleeding* OR *hepatic encephalopathy* OR *ascites* OR *portal hypertension, other* OR *cirrhosis, other.*
Portal hypertension	*Varices* OR *variceal bleeding* OR *hepatic encephalopathy* OR *ANZICS-APD GCS <15* OR *ascites* OR *portal hypertension, other* OR *ANZICS-APD flagged cirrhosis.*
Decompensated cirrhosis	*Variceal bleeding* OR *hepatic encephalopathy* OR *ANZICS-APD GCS <15* OR *ascites.*
Acute-on-chronic liver failure	*Decompensated cirrhosis* AND ≥2 of the following: (1) *brain failure* was defined as GCS < 15 on admission; (2) *respiratory failure* was defined as presence of assisted ventilation (invasive or non-invasive); (3) *renal failure* was defined as either requirement of renal replacement therapy, serum creatinine ≥ 350 µmol/L and/or 24-hour urine output < 500 mL; (4) *circulatory failure* was defined as mean arterial pressure < 60 mmHg and/or requirement of ionotropic support.

†ANZICS CORE – Adult Patient Database Data Dictionary, Version 6.1, April 2022. ANZICS Centre for Outcome and Resource Evaluation. Online: https://www.anzics.org/adult-patient-database-apd/

Supplementary Table 2. ICD-10 codes used to define complications of cirrhosis and cirrhosis aetiology.

Variable	ICD-10 codes
Varices	I850, I859, I864, I982, I983
Variceal bleeding	I850, I983
Hepatic encephalopathy	B190, G934
Ascites	R18
Portal hypertension, other	K766, K767
Cirrhosis, other	K703, K704, K717, K743, K744, K745, K746
Liver transplantation	Z944
Alcoholic liver disease	K70, F10, G312, G721, I426, K292, K852, K860, R780, T51, Y90, Z7141
Viral hepatitis	B17, N18, N19

Supplementary Table 3. APACHE-III diagnosis codes used to define ICU admission diagnosis categories and liver transplantation.

Variable	APACHE-III diagnosis codes
Liver failure	301
Upper GI bleeding	303, 305, 1403
Sepsis	201, 210, 212, 213, 404, 501, 502, 504, 504, 1102, 1301, 1406, 1409, 1412, 1904
Liver transplant	1407
